# A dataset for the effect of earthworm abundance and functional group diversity on plant litter decay and soil organic carbon level

**DOI:** 10.1016/j.dib.2020.105263

**Published:** 2020-02-08

**Authors:** Wei Huang, Grizelle González, Xiaoming Zou

**Affiliations:** a College of Biology and the Environment, Nanjing Forestry University, 159 Longpan Road, Nanjing 210037, Jiangsu, China; b Department of Environmental Sciences, College of Natural Sciences, University of Puerto Rico, P.O. Box 70377, San Juan, PR 00936-8377, USA; c International Institute of Tropical Forestry, USDA Forest Service, Jardín Botánico Sur, 1201 Calle Ceiba, Río Piedras, PR 00926-1119, USA

**Keywords:** Anecic worms, Endogeic worms, Epigeic worms, Forest floor mass, Litter decomposition, Soil carbon

## Abstract

This paper describes data of earthworm abundance and functional group diversity regulate plant litter decay and soil organic carbon (SOC) level in global terrestrial ecosystems. The data also describes the potential effect of vegetation types, litter quality, litterbag mesh size, soil C/N, soil aggregate size, experimental types and length of experimental time on earthworm induced plant litter and SOC decay. The data were collected from 69 studies published between 1985 and 2018, covering 340 observations. This data article is related to the paper “Earthworm Abundance and Functional Group Diversity Regulate Plant Litter Decay and Soil Organic Carbon Level: A Global Meta-analysis” [1].

**Table:** Specifications Table

Subject	Ecology, Soil Science
Specific subject area	Earthworm ecology, litter decomposition, soil carbon
Type of data	Table
How data were acquired	Systematic review of the literature
Data format	Raw
Parameters for data collection	We used three different combinations of keywords: earthworm and litter decomposition; earthworm and forest floor; earthworm and soil carbon.
Description of data collection	Data were collected from the ISI-Web of Science and Google Scholar.
Data source location	18 countries over five continents
Data accessibility	With the article
Related research article	Wei Huang, Grizelle Gonzalez, Xiaoming Zou, Earthworm Abundance and Functional Group Diversity Regulate Plant Litter Decay and Soil Organic Carbon Level: A Global Meta-analysis, Applied Soil Ecology, in press, https://doi.org/10.1016/j.apsoil.2019.103473. [1]

**Table:** 

**Value of the Data** • To date, no dataset has provided a comprehensive synthesis of existing experimental data about the effect of earthworms on litter decomposition and soil organic carbon (SOC) levels at global scale. • Data can be used to quantify the effect of earthworms on litter decomposition and SOC levels at global scale. • Data can be used to identify effects of earthworm functional group diversity, vegetation types, litter quality, litterbag mesh size, soil C/N, soil aggregate size, experiment types and length of experimental time on earthworm induced plant litter and SOC decay.

## 1 Data description

Data were extracted from peer-reviewed journal papers published between 1985 and 2018. Totally 340 observations from 69 studies were included. Detailed data are listed in Table 1, Table 2, Table 3, Table 4, Table 5, giving the following information: location, ecosystem, earthworm density, annual litter decomposition rate, earthworm function group, the response ratio (R), mean annual temperature, mean annual precipitation, experimental type, experimental duration, litter quality, forest floormass thickness and carbon stock, soil carbon concentration, soil C/N, soil aggregate size, and literature reference.

**Table 1:** Location, earthworm density, plant litter decomposition rate, and earthworm functional group in crop fields, tree plantations and forests worldwide for curve estimation.

Location	Ecosystem	Earthworm density (no./m^2^)	Annual litter decomposition rate (y^−1^)	Earthworm function group	Reference
Georgia, USA	Crop				
	Soy bean	176	1.67	Mixture	[3]
	Rye	176	1.45	Mixture	
Queensland, Australia	Sugarcane	199	1.88	Endogeic	[4]
	Plantation				
Dublin, Ireland	Salix	189	1.69	Mixture	[5]
Carlshead, UK	Short Rotation Forestry	152	0.91	Mixture	[6]
	Natural forest				
Puerto Rico, USA	Tabonuco (Upland)	45	1.47	Mixture	[7]
	Tabonuco (Riparian)	16	0.94	Mixture	
Anduze, France	Chestnut	86	1.50	Mixture	[8,9]
		86	0.55	Mixture	
		86	1.10	Mixture	
		86	0.64	Mixture	
		4	0.71	Anecic	
		4	0.56	Anecic	
		4	0.50	Anecic	
		4	0.37	Anecic	
		28	0.52	Mixture	
		28	0.52	Mixture	
		28	0.48	Mixture	
		28	0.25	Mixture	
Skane, Sweden	Beech	2.5	0.33	Epigeic	[10]
		39.8	0.60	Mixture	
		219.7	2.15	Mixture	
Hawaii, USA	Metrosiderus	21	0.37	Mixture	[11,12]
Puerto Rico, USA	Tabonuco (Control)	168.8	1.12	Mixture	[13]
	Tabonuco (Fertilization)	29.33	0.84	Endogeic	
	Subtropical lower montane rain forest (Control)	12	0.7	mixture	
	Subtropical lower montane rain forest (Fertilization)	19	1.49	Mixture	
Ontario, Canada	Sugar maple and American beech	67.675	0.39	Mixture	[14]
Colorado, USA	Aspen Forest	44.44	0.36	Mixture	[15]
		44.44	0.31	Mixture	
	Pine Forest	0.77	0.29	Epigeic	
		0.77	0.25	Epigeic	
New York State, USA	Sugar maple	79.6	1.05	Mixture	[16]
		26.5	0.51	Mixture	
		99.4	1.27	Mixture	
		26.1	0.6	Mixture	
	Oak	81.6	0.96	Mixture	
		26.4	0.53	Mixture	
		92.6	1.16	Mixture	
		21.5	0.63	Mixture

**Table 2:** The location, biome, mean annual temperature (MAT), mean annual precipitation (MAP), experimental type, experimental duration, earthworm functional group, earthworm numbers, litter quality for observations about the effects of earthworm on litter decomposition in the meta-analysis.

Location	Ecosystems	MAT (^o^C)	MAP (mm)	Experimental type	Experimental period (days)	Earthworm functional group	Litter type	Litter C/N	Litter bag mesh size (mm)	Effect size	References
Puerto Rico, USA	Pasture	22–26	3500	Field	365	Endogeic	Leaf	26	1	2.62	[17]
	Pasture	22–26	3500	Field	365	Endogeic	Root	101	1	1.10	
	Forest	20.8–24.5	3456	Field	365	Mixture	Leaf	32	1	1.22	
	Forest	20.8–24.5	3456	Field	365	Mixture	Root	101	1	1.12	
Maryland, USA	Forest (Tulip poplar Association-mature)			Field	240	Mixture	Leaf		10	2.29	[18]
				Field	240	Mixture	Leaf		1	1.12	
Anduze, France	Forest	11.9	1212	Field	760	Mixture	Leaf		5	2.33	[8] [9]
				Field	760	Mixture	Leaf		5	1.75	
				Field	760	Mixture	Leaf		5	2.42	
				Field	760	Mixture	Leaf		5	1.492	
Chicago, USA	Forest (Buckthorn)			Field	365		Leaf		4	33.76	[19]
				Field	365		Leaf		4	2.32	
				Field	365		Leaf		4	1.95	
				Field	365		Leaf		4	1.64	
	Forest (mesic)			Field	365		Leaf		4	9.81	
				Field	365		Leaf		4	3.73	
				Field	365		Leaf		4	2.33	
				Field	365		Leaf		4	2.56	
	Forest (maple)			Field	365		Leaf		4	2.79	
				Field	365		Leaf		4	0.77	
				Field	365		Leaf		4	1.73	
				Field	365		Leaf		4	0.94	
Ibadan, Nigeria	Crop			Lab	56	Epigeic	Leaf	10.1		2.53	[20]
				Field	56	Epigeic	Leaf	10.1		1.98	
New York, USA	Forest (Oak)		1000	Field	190	Mixture	Leaf		10	0.98	[21]
				Field	190	Mixture	Leaf		10	1.077	
	Forest (Sugar maple)			Field	190	Mixture	Leaf		10	1.027	
				Field	190	Mixture	Leaf		10	1.11	
	Forest (Oak)			Field	340	Mixture	Leaf		10	1.35	
				Field	340	Mixture	Leaf		10	1.51	
	Forest (Sugar maple)			Field	340	Mixture	Leaf		10	2.58	
				Field	340	Mixture	Leaf		10	1.53	
	Forest (Oak)			Field	540	Mixture	Leaf		10	1.68	
				Field	540	Mixture	Leaf		10	2.41	
	Forest (Sugar maple)			Field	540	Mixture	Leaf		10	1.56	
				Field	540	Mixture	Leaf		10	2.59	
Guangdong, China				Lab	126	Endogeic	Leaf			0.93	[22]
				Lab	126	Anecic	Leaf			1.42	
Baden Wurttemberg, Germany		14–22		Lab	63	Anecic	Leaf	17.3		1	[23]
		14–22		Lab	63	Anecic	Leaf	17.3		1.91	
		14–22		Lab	63	Anecic	Leaf	17.3		2.37	
Amazonas, Brazil		**24–31**		Lab	97	Endogeic	Leaf	27		0.95	[24]
				Lab	97	Endogeic	Leaf	32		1.03	
				Lab	97	Endogeic	Leaf	34		1.07	
				Lab	97	Endogeic	Leaf	42		1.04	
				Lab	97	Endogeic	Leaf	27		0.78	
				Lab	97	Endogeic	Leaf	32		0.89	
				Lab	97	Endogeic	Leaf	34		1.00	
				Lab	97	Endogeic	Leaf	42		0.98	
Tyrol, Austria		15 - 20		Lab	84	Endogeic	Leaf	34.7		0.96	[25]
				Lab	84	Epigeic	Leaf	34.7		1.00	
				Lab	84	Epigeic	Leaf	34.7		1.43	
				Lab	84	Mixture	Leaf	34.7		1.02	
				Lab	84	Mixture	Leaf	34.7		1.09	
				Lab	84	Epigeic	Leaf	34.7		1.12	
				Lab	84	Epigeic	Leaf	34.7		1.32	
				Lab	84	Endogeic	Leaf	34.7		1.11	
				Lab	84	Endogeic	Leaf	27.2		0.95	
				Lab	84	Epigeic	Leaf	27.2		1.04	
				Lab	84	Epigeic	Leaf	27.2		1.97	
				Lab	84	Mixture	Leaf	27.2		1.02	
				Lab	84	Mixture	Leaf	27.2		1.31	
				Lab	84	Epigeic	Leaf	27.2		1.25	
				Lab	84	Epigeic	Leaf	27.2		2.05	
				Lab	84	Endogeic	Leaf	27.2		1.56	
Wisconsin, USA	Forest			Field	123	Anecic	Leaf			4.62	[26]
Minnesota, USA	Temperate deciduous forest	18		Lab	42	Anecic	Leaf			1.50	[27]
		18		Lab	42	Epigeic	Leaf			2.35	
		18		Lab	42	Mixture	Leaf			2.80	
				Field	82	Anecic	Leaf			1.06	
				Field	82	Epigeic	Leaf			1.47	
				Field	82	Mixture	Leaf			1.37	
Tyrol, Austria		15		Lab	28	Epigeic	Leaf			1.07	[28]
		15		Lab	28	Epigeic	Leaf			1.11	
		15		Lab	28	Epigeic	Leaf			1.17	
		15		Lab	28	Epigeic	Leaf			1.21	
Bechstedt, Germany		15–20		Lab	56	Anecic	Leaf			2.12	[29]
				Lab	56	Anecic	Leaf			2.68	
				Lab	56	Anecic	Leaf			3.15	
				Lab	56	Anecic	Leaf			3.26	
				Lab	56	Anecic	Leaf			2.67	
				Lab	56	Anecic	Leaf			4.00	
				Lab	56	Anecic	Leaf			13.28	
				Lab	56	Anecic	Leaf			6.28	
				Lab	56	Anecic	Leaf			1.34	
				Lab	56	Anecic	Leaf			1.06	
				Lab	56	Anecic	Leaf			35.85	
				Lab	56	Anecic	Leaf			2.15	
				Lab	56	Anecic	Leaf			5.95	
				Lab	56	Anecic	Leaf			1.33	
				Lab	56	Anecic	Leaf			2.18	
				Lab	56	Anecic	Leaf			4.72	
				Lab	56	Anecic	Leaf			9.63	
				Lab	56	Anecic	Leaf			1.16	
				Lab	56	Anecic	Leaf			1.20	
				Lab	56	Anecic	Leaf			1.56	
				Lab	56	Anecic	Leaf			1.80	
				Lab	56	Anecic	Leaf			3.34	
				Lab	56	Anecic	Leaf			11.36	
				Lab	56	Anecic	Leaf			6.97	
				Lab	56	Anecic	Leaf			12.36	
Puerto Rico, USA				Lab	22	Mixture	Leaf			2.10	[30]
Hampshire, UK	Short rotation forestry	11.2	630	Field	365	Mixture	Leaf	32.5		2.26	[31]
				Field	365	Mixture	Leaf	39.5		1.51	
Carlshead, UK	Short rotation forestry	9	1000	Field	365	Mixture	Leaf	39.5	5	5.28	[6]
				Field	365	Mixture	Leaf	52	5	8.15	
				Field	365	Mixture	Leaf	33	5	12.44	
				Field	365	Mixture	Leaf	32.5	5	10.41	
				Field	261	Mixture	Leaf	18.2	5	17.56	
Kaserstattalm, Austria		9–17		Lab	120	Epigeic	Leaf			1.35	[32]
				Lab	120	Epigeic	Leaf			1.07	
				Lab	120	Epigeic	Leaf			2.50	
Gottingen, Germany		18		Lab	90	Epigeic	Leaf			1.24	[33]

**Table 3:** Location, earthworm density, and forest floormass thickness and carbon stock in forests worldwide for curve estimation.

Location	Earthworm density (no./m^2^)	Forest floormass		References
		Thickness (cm)	Carbon stock (g/m^2^)	
Minnesota, USA	592.00	0.60		[34]
Minnesota, USA	821.47	1.14		[35]
Ontario, Canada	99.50	2.70		[36]
Alberta, Canada	622.72	4.19		[37]
	181.59	3.66		
	108.14	3.57		
	136.42	3.49		
	162.75	2.64		
	214.18	1.01		
	196.08	0.97		
	623.02	0.20		
	458.67	0.12		
	661.73	0.04		
Maryland, USA	212.00	1.00	116.00	[38]
Maryland, USA	38.00	6.25		[39]
Michigan, USA	9.10		895.60	[40]
	247.80		316.20	
New York State, USA	106.30		211.20	[41]
	76.83		70.40	
New York State, USA	150.00		196.34	[42]
	89.20		295.39	
Puerto Rico, USA	32.67		785.10	[43]
	56.00		406.40	
	8.76		563.90	
Jilin, China	780	1.0		[44]
	336	2.5		
	153	2.0		
	52	1.5		
Yunan, China	28.5	1.5		[45]
	12.35	0.5		
	7.5	1

**Table 4:** Location, earthworm density, and mineral soil carbon concentration in 12 sites of crop fields, pasture, and forests worldwide used for curve estimation.

Location	Ecosystems	Earthworm density (no./m^2^)	Soil depth (cm)	Soil organic C concentration (%)	Earthworm functional group	References
Ohio, USA	Crop					
	Corn-soybean	17.9	0–10	16.1	Mixture	[46]
			10–20	12.4		
			20–30	12.3		
			30–40	8.8		
Jiangsu, China	Rice–wheat	30	0–20	8.04	Anecic	[47]
				9.09		
Timiş, Romania	Wheat-soybean-maize-barley	9.33		2.26		[48]
		14.76		2.16		
		9.33		2.16		
		13.33		2.10		
		26.67		2.53		
Tennessee, USA	Rotation		0–15			[49]
	Corn -soybean	46.05		1.2	Mixture	
	Continuous Soybean	52.85		1.4	Mixture	
	Continuous Corn	40.5		1.0	Mixture	
	Bio-cover					
	Fallow	45.8		1.1	Mixture	
	Hair vetch	75.5		1.1	Mixture	
	Poultry litter	27.35		1.3	Mixture	
	Wheat	36.75		1.1	Mixture	
Hawaii, USA	Eucalypt	12	0–25	7.55	Endogeic	[50]
		151		8.52	Endogeic	
		154		8.80	Endogeic	
		398		9.86	Endogeic	
Eifel, Germany	Four crop rotation (rape, winter wheat, winter barley, and spring barley)	119.3	0–10	1.56	Mixture	[51]
			10–20	1.52		
			20–30	0.87		
		113.3	0–10	1.79	Mixture	
			10–20	1.22		
			20–30	0.75		
		160	0–10	1.94	Mixture	
			10–20	1.23		
			20–30	0.74		
		132.7	0–10	1.71	Mixture	
			10–20	1.14		
			20–30	0.68		
		157.3	0–10	1.75	Mixture	
			10–20	1.15		
			20–30	0.67		
Karnataka, India	Agricultural fields (rice, nuts, and banana)	485.14	0–30	4.94	Mixture	[52]
KwaZuluNatal midlands, South Africa	Ryegrass	158.82	0–10	3.74	Mixture	[53]
	Maize	49.27		3.12	Mixture	
	Sugarcane	25.74		2.56	Epigeic	
	Ryegrass	76.53		3.21	Mixture	
	Maize	45.79		2.68	Mixture	
	Sugarcane	164.69		3.06	Epigeic	
Victoria, Australia	Crop	21.00	0–7.5	0.93		[54]
		46.00		0.94		
		50.00		0.96		
	Pasture					
New Zealand		637	0–5	3.98	Mixture	[55]
			5–10	4.10		
			10–18	3.30		
			18–26	3.20		
KwaZuluNatal midlands, South Africa	Kikuyu grass	236.03	0–10	7.58	Mixture	[53]
	Native grassland	6.08		5.79		
	Kikuyu grass	303.34		8.07	Mixture	
	Forest					
New York, USA	Forest	106	0–5	5.75	Mixture	[39,40]
			5–10	2.63		
			10–15	1.65		
			15–20	1.43		
		76	0–5	6.97	Mixture	
			5–10	4.12		
			10–15	1.93		
			15–20	1.71		
Honduras Karnataka, India	Forest	37.89	0–15	3.59	Endogeic	[56]
	Forest	561.06	0–30	5.24	Mixture	[52]
KwaZuluNatal midlands, South Africa	Gum forest	60.29	0–10	3.53	Endogeic	[53]
	Pine forest	18.38		4.45	Mixture	
	Gum forest	60.97		5.62	Endogeic	
	Pine forest	19.91		5.51	Mixture	
Hawaii, USA	Eucalypt	173	0–25	8.90	Mixture	[50]
		147		9.43	Mixture

**Table 5:** The location, biome, MAT, MAP, experimental type, earthworm functional group, earthworm number, soil depth, soil C/N and soil aggregate size for observations about the effects of earthworm on soil organic carbon levels in the meta-analysis.

Location	Ecosystems	MAT (^o^C)	MAP (mm)	Experimental type	Earthworm functional group	Soil depth (cm)	Experimental period	Soil C/N	Soil aggregate size	Effect size of soil organic carbon	References
New York, USA	Forest		900	Field	Mixture	0 - 5	730	13.3		0.62	[41]
					Mixture	5 - 10	730	11.6		0.81	
					Mixture	10 - 15	730	10.1		0.62	
					Mixture	15 - 20	730	10.0		0.65	
					Mixture	0 - 5	730			0.75	
					Mixture	5 - 10	730			1.27	
					Mixture	10 - 15	730			0.72	
					Mixture	15 - 20	730			0.78	
New York, USA	Forest		900	Field	Mixture	0 - 5	730			0.86	[57]
					Mixture	5 - 10	730			1.10	
					Mixture	10 - 15	730			0.62	
					Mixture	15 - 20	730			0.72	
New Zealand	Pasture	12.2	1050	Field	Anecic	0 - 5	10950			0.82	[55]
						5 - 10	10950			0.75	
						10 - 18	10950			0.58	
						18 - 26	10950			0.82	
						0 - 5	7300			0.98	
						5 - 10	7300			1.06	
						10 - 18	7300			1.05	
						18 - 26	7300			1.24	
New York, USA	Sugar maple		980	Field		0 - 3		18.73		1.34	[42]
						3 - 6		17.53		1.14	
						6 - 9		16.80		1.08	
						9 - 12		15.84		0.96	
						0 - 3		13.59		1.17	
						3 - 6		11.83		0.99	
						6 - 9		11.59		1.05	
						9 - 12		11.18		0.95	
Cumbria, UK		15		Lab		0 - 8	110			1.06	[58]
Tennessee, USA		20		Lab	Endogeic		26		>250	2.05	[59]
					Endogeic		26		53–250	0.78	
					Endogeic		26		<53	1.30	
					Epigeic		26		>250	3.60	
					Epigeic		26		53–250	0.96	
					Epigeic		26		<53	1.13	
Ohio, USA	Corn-soybean			Field	Mixture	0 - 10	1075			1.11	[46]
					Mixture	10 - 20	1075			1.19	
					Mixture	20 - 30	1075			1.01	
					Mixture	30 - 40	1075			1.02	
Jiangsu, China	Rice–wheat	16	1106	Field	Anecic	0 - 20	2555	8.30		1.02	[47]
							2555			1.02	
Quebec, Canada	Hardwood forest	6.2	1058	Field		0–10		14.00		1.56	[60]
						10–20		13.30		1.50	
Xishuangbanna , China	Rubber plantation	21.8	1493	Field	Endogeic	0–5	600	11.80		0.94	[61]
						5–15	600	11.80		1.05	
						0–5	600	11.80		0.72	
						5–15	600	11.80		1.45	
Congo, Brail	Savanna				Endogeic	0–10				0.67	[62]
						10–20				1.31	
						20–30				1.00	
Georgia, USA				Lab	Endogeic		20		>2000	3.42	[63]
							20		250–2000	0.52	
Georgia, USA				Lab	Endogeic		20		>2000	3.12	[64]
							20		250–2000	0.78	
							20		53–250	0.71	
							20		<53	0.61	
Great Smoky Mountains National Park, USA		18		Lab	Epigeic		23			0.92	[65]
							23			0.89	
							23		>2000	10.25	
							23		>2000	5.32	
							23		250–2000	0.59	
							23		250–2000	0.80	
							23		53–250	0.08	
							23		53–250	0.66	
Trier, Germany		15		Lab	Mixture		42	14.88		1.01	[66]
							42	14.31		1.06	
							42	15.25		0.99	
							42	15.25		1.03	
Georgia, USA				Lab	Endogeic	0–3.5	37			1.03	[67]
					Epigeic	3.5–7	37			1.09	
					Endogeic	0–3.5	37			0.98	
					Epigeic	3.5–7	37			1.08	
Alberta, Canada				Lab	Epigeic	1–4	28			1.03	[68]
						1–4	56			0.89	
						1–4	84			0.96	
						1–4	28			0.73	
						1–4	56			0.89	
						1–4	84			0.70	
						4–7	28			0.94	
						4–7	56			0.90	
						4–7	84			1.00	
						4–7	28			0.79	
						4–7	56			1.00	
						4–7	84			0.68	
						>7	28			1.16	
						>7	56			1.29	
						>7	84			1.04	
						>7	28			1.60	
						>7	56			1.23	
						>7	84			1.94	
Jilin, China		18		Lab		0–2.5	30			0.95	[69]
						0–2.5	30			1.12	
						0–2.5	30			0.94	
						0–2.5	30			1.18	
						2.5–5	30			1.03	
						2.5–5	30			0.77	
						2.5–5	30			0.95	
						2.5–5	30			1.14	
Hubei, China		25±2		Lab	Anecic		40			0.96	[70]
							40			0.77	
							40		<250	1.10	
							40		250–1000	0.79	
							40		1000–2000	1.21	
							40		>2000	1.19	
Jinlin, China		20		Lab	compost		18	13.04		1.04	[71]
							18	13.04		1.15	
							18	13.04		1.04	
							35	14.09		1.12	
							35	14.09		1.10	
							35	14.09		1.08	
Puerto Rico, USA				Lab	Anecic		22			0.98	[30]
					Endogeic		22			1.01	
					Endogeic		22			0.94	
					Mixture		22			0.99	
					Mixture		22			0.97	
					Mixture		22			0.97	
					Mixture		22			0.97	
Hanoi, Vietnam		15–25		Lab	Endogeic		365			1.02	[72]
					Endogeic		365			0.82	
					Endogeic		365			0.81	

## 2 Experimental design, materials, and methods

A data set was compiled using literature search of peer-reviewed publications about the effects of earthworms on litter decomposition or SOC from the ISI-Web of Science and Google Scholar research database. We used three different combinations of keywords: earthworm and litter decomposition; earthworm and forest floor; earthworm and soil carbon. A total of 69 studies published between 1985 and 2018 were found (Table 1, Table 2, Table 3, Table 4, Table 5). An Engauge Digitizer (Free Software Foundation, Inc., Boston, MA, United States of America) was used to extract numerical values from figures in selected articles in which data were graphically presented.

For Table 1, we included studies that reported earthworm density and litter decomposition/decay rate; 40 observations from 13 studies were found. For Table 3, we included studies that reported earthworm density and forest floor thickness or carbon stock; 32 observations from 12 studies were found. For Table 4, we included studies that reported earthworm density and soil carbon content (%, g C/kg soil or mg C/g soil); 70 observations from 12 studies were found. For Table 1, Table 3, Table 4, we included studies that reflected earthworm density under field conditions (i.e. earthworms were not reduced or added), and plant litter from the vegetation currently under the experimental sites so that these observations can reflect the balance between earthworm density and turnover of plant litter, SOC under field conditions.

To be included in the meta-analysis, the paper had to report the means, standard deviation (SDs) and replicate numbers of litter percent mass loss or SOC for the control treatment (C, with no earthworms or reduced earthworm number) and the experimental treatment (E, with earthworms or earthworm number do not reduce). For studies that did not report SD or standard error (SE), we conservatively estimated SD values as 150% of the average variance across the dataset [2]. To evaluate the significance of the earthworm-induced effect on litter decomposition, 113 observations from 20 studies were found (Table 2). For the magnitude of the earthworm-induced effect on SOC content, 120 observations from 22 studies were found (Table 5). Because most of the studies do not report soil bulk density, we therefore converted SOC stocks with known bulk density (20 observations) to SOC concentrations. Besides earthworm functional groups, other details of experimental conditions were also specified in our analyses. We included studies that reported climate, vegetation types (naturally-grown forest, plantation, pastureland and crop), litter quality (litter C/N ratio and leaf versus root litter), litterbag mesh size, time length of experiment, soil depth, soil aggregate size, soil C/N ratio and experimental types (field versus laboratory). These parameters were the controlling factors that we considered for the earthworm effect on litter decay and SOC. The magnitude of the earthworm-induced effect on litter decay and SOC were calculated as the response ratio (R), R = E/C, where E and C are the means of experimental and control treatments, respectively.
